# The multi-level paths from age diversity to organizational citizenship behaviors: could leader/team-member exchange be answers that benefit the paths?

**DOI:** 10.3389/fpsyg.2025.1413940

**Published:** 2025-03-03

**Authors:** Linyuan Zhang

**Affiliations:** Shandong Women’s University, Jinan, China

**Keywords:** age diversity, group organizational citizenship behavior (GOCB), organizational citizenship behavior (OCB), leader-member exchange (LMX), team-member exchange (TMX)

## Abstract

**Introduction:**

Organizational citizenship behavior (OCB) plays a crucial role in fostering the continuous growth and development of organizations. This essay aligns with the current labor force structure changes resulting from population aging, focusing on exploring the relationship between age diversity and multi-level OCB.

**Methods:**

A multi-level moderation model was employed to collect data from 882 employees across 87 groups of Chinese state-owned enterprises. Linear regression and hierarchical linear modeling (HLM) were used to test the hypotheses.

**Results:**

The findings of this study indicate that (1) leader-member exchange (LMX) moderates the negative effect of age diversity on group-level organizational citizenship behavior (GOCB); (2) team-member exchange (TMX) moderates the negative relationships between age diversity and individual-level organizational citizenship behavior toward organizations (OCBO) and organizational citizenship behavior toward individuals (OCBI).

**Conclusion:**

The empirical study carries substantial implications for future discourse on human resource practices (HRPs) and research pertaining to population aging within organizational contexts.

## Introduction

The age composition of most economies is currently undergoing a significant transformation (Nagarajan et al., 2019; United Nations, Department of Economic and Social Affairs, Population Division, 2015; Wheaton and Crimmins, 2013). This transformation is driven by the simultaneous increase in aging populations and extended longevity and careers, as well as delayed retirement (Brown and Guttmann, 2017; Loretto and Vickerstaff, 2013), resulting in rapid growth in age diversity within the workforce (Wegge and Meyer, 2020; Profili et al., 2017; Harrison and Klein, 2007). The contemporary workplace is characterized by the simultaneous employment of multiple generations (Meister and Willyerd, 2010), resulting in noticeable generational gaps and an unprecedented level of age diversity (Levi, 2017; Ng and Parry, 2016). Consequently, it is imperative for organizations to ensure the effective management of workgroups consisting of individuals from diverse age groups. Organizations and managers are expected to possess reliable foresight and employ strategies that promote optimal collaboration and guidance within workgroups with diverse age groups (Kunze and Bruch, 2010).

However, despite the increasing scholarly focus on comprehending the implications of age diversity (e.g., Scheuer and Loughlin, 2021; Seong and Hong, 2018), limited attention has been given to understanding how age diversity affects outcomes or influences diverse outcomes within an organization (Seong and Hong, 2018). The effects of age diversity in the workplace have produced inconsistent findings (van Knippenberg and Schippers, 2007; van Knippenberg et al., 2004). Some studies indicate that a high level of age diversity negatively impacts outcomes because of group divisions caused by intergroup biases (e.g., Jukka, 2021; van Dijk et al., 2012). Conversely, several studies on age diversity assert that it can be considered a competitive advantage (Li et al., 2011) by providing diverse information and a wealth of perspectives (Luksyte et al., 2022). In addition, there is evidence demonstrating a non-linear impact of age diversity (e.g., Seong and Hong, 2018), as well as insignificant effects (e.g., Schneid et al., 2016). High levels of age diversity do not necessarily exert a negative influence on outcomes. In line with the categorization-elaboration model (CEM; van Knippenberg et al., 2004), each type of diversity may elicit both social categorization processes and information/decision-making processes albeit with varying degrees. Therefore, acknowledging the indispensable role played by contextual conditions in moderating these relationships is crucial. Contextual conditions refer to “situational opportunities or constraints that affect the occurrence and meaning of organizational behavior as well as functional relationships between variables” (Johns, 2006, p. 386). Previous research suggests that the impact of diversity depends on the organizational context and the nature of diversity itself (Mamman et al., 2012). Thus, this study focuses on specific contextual conditions to examine the relationships between age diversity and its outcomes, such as organizational citizenship behavior (OCB)/group organizational citizenship behavior (GOCB).

OCB encompasses discretionary actions and employee commitment aimed at optimizing operational efficiency within organizations, thereby making a significant contribution to organizational success (Podsakoff et al., 2009). Consistent with the argument proposed by Organ et al. (2006), contemporary organizations rely more on employees’ voluntary contributions and extra-role behaviors to achieve goals and enhance performance, rather than solely relying on required or enforced behaviors dictated by formal systems. Extra-role behavior plays a critical role in organizational effectiveness because managers cannot anticipate all contingencies that may arise (Morrison and Phelps, 1999). Therefore, the concept of OCB effectively captures the benefits derived from having a diverse workforce. Recognizing age diversity’s influence on OCB is crucial for firms’ success and productivity.

Notably, although the notion of OCB initially emerged at the individual level, criticisms have been raised against analyses that solely focus on individual-level OCB as they “fall short of fully capturing OCB phenomenon” (Somech and Drach-Zahavy, 2004, p. 292). Recently, scholars have shifted their attention toward conceptualizing OCB as a group-level phenomenon (Seong and Hong, 2018; Choi and Sy, 2010). That is, GOCB represents an attribute that is specific to the group, and is conceptually and empirically distinct from individual-level OCB (Bommer et al., 2007). In order to conduct a more comprehensive investigation into the effects of age diversity on its outcomes, this paper simultaneously uses both GOCB and individual-level OCB as outcome variables to examine the influences of age diversity.

This study establishes a conceptual model of the moderating factors that influence the relationships between age diversity and its group- and individual-level outcomes. These moderating factors have been overlooked or not adequately considered in previous diversity research. In terms of moderation variables for the relationships between age diversity and GOCB/OCB, this paper focuses on leader-member exchange (LMX) and team-member exchange (TMX). Because leaders and co-workers are considered vital partners within a workgroup (Taylor et al., 2004). In the workplace, the vertical and horizontal exchange relationships among individuals within a group serve as valuable social contexts. Correlational research primarily focuses on examining the effect of exchange quality on outcomes (Liden et al., 1997). A favorable exchange relationship is closely related to positive work outputs, attitudes, and behaviors (Halbesleben, 2010).

Previous studies have found a positive association between LMX and OCB, indicating that employees are more likely to engage in OCB when they perceive a high-quality relationship with their leaders (e.g., Mitchell et al., 2024; Ilies et al., 2007). By bridging the individual beliefs and identities of followers with the collective beliefs and identities of the groups, LMX can facilitate a transition toward group-oriented organizational citizenship behavior (i.e., GOCB). Given that effective leadership has been proved to minimize the negative effects and maximize the positive effects of group diversity on group outcomes (Seong and Hong, 2018; Shin and Zhou, 2007), this investigation proposes that LMX is likely to mitigate the detrimental effects of age diversity on GOCB.

Additionally, this paper has chosen TMX as the moderating factor in the relationships between age diversity and individual outcomes. The harmonious relationship resulting from high levels of TMX contributes to mitigating intergroup prejudices caused by age heterogeneity (Scheuer and Loughlin, 2019). Hence, this research states that TMX may alleviate the detrimental effect of age diversity on OCB at the individual level.

In summary, this article serves two purposes. First, this paper adopts a comprehensive and holistic approach to conduct multi-level analyses of an aging labor force by examining the relationship between age diversity and its outcomes at both group and individual levels, such as GOCB and OCB. Second, this study analyzes contextual factors, such as LMX and TMX to determine whether they moderate the effects of age diversity on both group and individual outcomes in order to advance workplace aging research.

## Literature review and hypothesis development

### Age diversity

The phenomenon of population aging is currently unfolding with undeniable force as illustrated by the impactful terms “demographic time bomb” (Tempest et al., 2002, p. 487) and “age quake” (Tempest et al., 2002, p. 489). The proportion of older individuals has experienced a significant surge (Loretto and Vickerstaff, 2013; Vickerstaff, 2010), which when combined with the potential increase in the legal retirement age (Loretto et al., 2013; Kunze and Bruch, 2010), can have profound implications for both organizational and national workforces. There has been a discernible diversification of age within workforce environments (e.g., Nagarajan et al., 2019; Stone and Tetrick, 2013).

In accordance with the CEM (van Knippenberg et al., 2004), the influence of group diversity on groups is exerted through both social categorization processes and information/decision-making processes. Specifically, social categorization processes point out that individuals tend to classify others as either in-group or out-group members based on visible attributes such as age, subjectively. People have a tendency to identify with similar others as part of an in-group and perceive dissimilar others as part of an out-group (van Knippenberg et al., 2004). Intergroup bias may arise when individuals perceive dissimilar others as a threat to maintaining a positive self-image (Scheuer and Loughlin, 2021). In situations where age differences are noticeable within groups, these discrepancies may result in intergroup biases and hostile intergroup relations (van Knippenberg and Mell, 2016; van Knippenberg and Schippers, 2007; van Knippenberg et al., 2004). Nevertheless, when age-diverse work groups prioritize exchanging and sharing information and knowledge (i.e., information/decision-making processes) rather than excessively emphasizing social categorization processes, they can benefit from diversity. Thus, in order to reduce the influence of social categorization processes and improve the explanatory power of information/decision-making effects on the relationship between age diversity and its outcomes, this article integrates potential moderators into the theoretical framework. The theoretical model of this research incorporates LMX as a moderator in the relationship between age diversity and GOCB, as well as TMX moderating the pathway from age diversity to OCB.

### GOCB and OCB

The concept of OCB, as defined by Organ (1988), refers to voluntary individual behaviors that can enhance organizational effectiveness even if they are not directly or explicitly recognized by the formal reward system. In order to differentiate OCB from other concepts, scholars have identified various dimensions for it. These multidimensional delineations categorize OCB into altruism, civic virtue, courtesy, conscientiousness, and sportsmanship (Podsakoff et al., 1990). Moreover, researchers also categorize OCB into two primary second-order dimensions: individual-level organizational citizenship behavior toward organizations (OCBO) and individual-level organizational citizenship behavior toward individuals (OCBI), based on the beneficiary protagonist (Williams and Anderson, 1991). In the light of Williams and Anderson (1991), OCBO involves “benefit the organization in general” (Williams and Anderson, 1991, p. 601) such as civic virtue, sportsmanship, and conscientiousness (Graham, 1991). In contrast, OCBI means actions that “immediately benefit specific individuals and indirectly through this means contribute to the organization” (Williams and Anderson, 1991, p. 602). Altruism and courtesy are among the most common forms of OCBI (Borman and Motowidlo, 1993).

Organ (1988) indicates that “most OCB action, taken singly, would not make a dent in the overall performance of the organization … any single occurrence of it usually is modest or trivial” (p. 8). In other words, there seems to be little effect from individual-level OCB on higher-level outcomes. Consequently, the mechanisms underlying individual-level OCB should become apparent at the group level through significant group mechanisms. This perspective supports Chan’s (1998) claim that most organizational phenomena are “inherently multilevel as opposed to occurring at a single level or in a level vacuum” (p. 234). As a result, there has been an emergence and growing attention toward GOCB as it relates to how employees collectively engage in OCB within their workgroups (Chen et al., 2002).

In order to clarify the emergence of lower-level elements into higher-level constructs, it is imperative to conceptually differentiate between group-level OCB (i.e., GOCB) and individual-level OCB. Similar to other multi-level constructs such as self-efficacy and collective efficacy, they share a common content domain but represent specific organizational phenomena with different referents (individual vs. group). This shift in referent “results in a new form of original construct that is conceptually distinct from the original form” (Chan, 1998, p.239). Building upon the referent-shift consensus model (Chan, 1998), the conceptualization of GOCB implies that it is “not the aggregate to the unit of OCB ratings of individuals toward other members of group, where the individual is the referent” (Chen et al., 2005, p. 275). Instead, GOCB represents a construct oriented toward groups derived from complex and reciprocal interactions among group members (Marotto et al., 2010; Morgeson and Hofmann, 1999).

### Hypothesis development

Based on van Knippenberg et al.’s (2004) definition, age diversity can be defined as the differences in individuals’ age attributes that may lead to perceiving others as different from oneself. Age diversity is heavily influenced by social categorization processes (e.g., Fiske, 2017; North and Fiske, 2015), which can be disruptive due to the presence of age stereotypes and intergroup bias.

GOCB refers to the normative level of OCB exhibited within a workgroup (Ehrhart, 2004), representing the collective behavior of individuals for organizational development (Borman and Motowidlo, 1997). OCB is described as discretionary employee behavior that is important for an organization’s long-term viability (Takeuchi et al., 2015). According to Williams and Anderson (1991), OCB can be differentiated based on its beneficiary or target as OCBO and OCBI, respectively. These terms are widely used in literature (Podsakoff et al., 2009; Organ et al., 2006). OCBO encompasses behaviors that generally benefit the entire organization while OCBI refers to behaviors that directly benefit other individuals and indirectly benefit the organization itself (Williams and Anderson, 1991). Based on the social categorization (Turner, 1987) and social identity theories (Tajfel, 1982), group members tend to engage in stereotyping individuals who belong to different age categories within heterogeneous units. This leads to biases against outgroup individuals who are considered less trustworthy, less capable, and less cooperative compared to ingroup members (Hogg and Terry, 2000). As a result, the interaction among dissimilar age employees may be diminished. Chattopadhyay (1999), and Messick and Brewer (1983) indicate that decreased interaction can result in a lack of trust which ultimately leads to lower levels of OCB (Mamman et al., 2012). This aligns with the argument made by Northcraft et al. (1995), which states that heterogeneity within organizations fosters bias and stereotypes among individuals thereby posing challenges for group identification and OCB.

Furthermore, the presence of power imbalances among employees rooted in demographic traits (e.g., age) may hinder the establishment of reciprocal norms (Gouldner, 1960). When influential individuals such as elders impose inequitable exchange relationships on others, this obstacle can be exacerbated (Choi, 2009). In this scenario, negative interpersonal perceptions are likely to emerge and undermine both GOCB and OCB. Choi and Sy (2010) discuss that age faultlines influence relationship conflict in groups, which is considered a detrimental predictor of GOCB (Seong and Hong, 2018). Age diversity has an adverse impact on GOCB due to the emergence of negative interpersonal perceptions, such as conflicts (e.g., Pelled et al., 1999). Thus, the author proposes the following hypotheses:

*H1:* Age diversity will negatively affect GOCB.

*H2a:* Age diversity will negatively affect OCBO.

*H2b:* Age diversity will negatively affect OCBI.

Since age differences among group members are inevitable, which may lead to negative outcomes (Leonard and Straus, 1996) such as low levels of GOCB and OCB, it is crucial to consider the potential contextual factors that can help mitigate these risks. According to Hackman (1992), the immediate working group serves as the most prominent social context for individuals in the workplace. The group is characterized by relational phenomena described by Kidwell et al. (1997), and comprises leaders and workmates (Taylor et al., 2004). Group managers and co-workers play a significant role in maintaining frequent contact and interaction with members during day-to-day activities within the workgroup setting (Taylor et al., 2004). As such, this study aims to examine the associations between age diversity and GOCB/OCB within the context of relationship-related LMX and TMX, described as “the reciprocal exchange relationships of an employee with the leader and other members” (Seong and Choi, 2019, p. 132). The essence of such relationships lies in the establishment of mutual trust, respect, and commitment (Graen and Uhl-Bien, 1995; Seers, 1989).

Previous studies have consistently emphasized the pivotal role of leaders as significant providers of sensory information in interpreting and framing organizational reality, thereby shaping employees’ sensemaking processes (Maitlis and Lawrence, 2007; Bartunek et al., 1999). Due to their legitimate authority and control over substantial resources (e.g., norms management and scheduling) and outcomes (e.g., performance appraisal and reward systems), superiors exert considerable effects on their subordinates (Dineen et al., 2006; Eisenberger et al., 2002). In essence, as the “managers of meanings” (Staw and Sutton, 1993), group leaders play a crucial role in shaping the perceptions of key organizational and group exchange actors (e.g., employees). One evident way for leaders to exert influence on these perceptions is by impacting interpersonal relationships within a specific group.

According to Blau’s (1964) perspective, social exchange is a relational dynamic that relies on unspecified future obligations. This type of exchange requires trust between the parties involved, and each party believes that the other will fulfill their commitments in the long term (Holmes, 1981). In groups characterized by high levels of age diversity, subgroups tend to exhibit heightened sensitivity toward breaches of the psychological contract when they find themselves in vulnerable positions within the group. For example, the failure to provide adequate support and effectively implement it would be perceived as a violation of the relational psychological contract. Such violations have the potential to erode group trust, commitment, loyalty, and subsequently diminish constructive behaviors toward the group. LMX refers to the quality of a follower’s relationship with his/her leader (Graen and Uhl-Bien, 1995). It explores how superiors effectively manage and cultivate different exchange relationships with their followers over time (Farouk, 2002). When leaders establish close relationships characterized by high-quality LMX with certain followers (Graen and Uhl-Bien, 1995), subgroup members are likely to perceive increased attention and support from their superiors. This can motivate an exchange featured by elevated levels of trust, respect, liking, and satisfaction (Gerstner and Day, 1997), thereby contributing to identity formation and the improvement of cohesion within the group. As such, the enhancement of constructive behaviors such as GOCB becomes feasible (e.g., Shin and Choi, 2010).

Moreover, groups with a high level of LMX benefit from delegation, responsibility, allocation, and autonomy. These factors enable group members to have greater decision-making latitude and contribute meaningfully to the collective goals of the group (Gomez and Rosen, 2001). Under such circumstances, collective beliefs emerge among members wherein they feel well-integrated within the group and trust each other’s commitment toward shared interests despite age differences. As a result, individuals tend to engage in collective extra-role actions. That is, a high LMX can mitigate or eliminate adverse effects of age diversity on GOCB. Thus, the author proposes the following hypothesis:

*H3:* LMX will moderate the negative effect of age diversity on GOCB, such that the negative effect of age diversity on GOCB will be mitigated when LMX is high.

Due to frequent interactions and extensive time spent together on workdays, colleagues constitute an immediate social environment for employees (LePine and van Dyne, 2001). Consequently, the interplay between age diversity and daily social exchanges within this immediate social environment, referred to as TMX in this article, may reveal the potential motivational mechanism affecting individual OCB.

TMX refers to the quality of reciprocal exchange relationships that exist between an employee and other members (Seers, 1989). Research has provided evidence supporting the association between social exchange assumptions and employee behaviors (e.g., Eisenberger et al., 2001; Orpen, 1994). When individuals identify and join a group marked by high TMX that aligns with their preferences, they can perceive and experience strong emotional connections, and recognize compatibility within their group membership (Chiniara and Bentein, 2017). In high TMX group, individuals transcend age-based social categorization by considering every group member, including themselves, as integral parts of the collective (De Janasz et al., 2015). Such harmonious group relationships contribute to alleviating intergroup biases in age-diverse groups (Scheuer and Loughlin, 2019), thereby reducing the negative effects of age diversity on its outcomes. Based on the social exchange theory (Blau, 1964), group employees are motivated to concentrate on fulfilling additional responsibilities in order to receive attention, recognition, and rewards from the group and other performers engaged in OCBO. Employees are expected to act in the best interest of the organization by surpassing established norms. Despite age differences among members, each individual is encouraged to engage in extra-role behaviors aimed at improving organizational functioning (i.e., OCBO; Takeuchi et al., 2015; Organ and Paine, 1999). In other words, high TMX will mitigate the detrimental effects of age diversity on OCBO.

In addition, individuals belonging to a high TMX group are likely to exhibit more positive attention and warmth toward their colleagues, leading to a congenial and pleasant work environment even in the presence of significant age diversity. This reduces the likelihood of activating age-based stereotypes among group members. Individuals in a high TMX setting tend to prioritize the kindness demonstrated by their co-workers, rather than fixating on demographic dissimilarities. Consequently, this may lead to a reduction in the detrimental social categorization processes caused by age diversity. In line with the social exchange theory (Blau, 1964), individuals are inclined to reciprocate the goodwill received from others by providing additional assistance to their colleagues. (i.e., OCBI; Williams and Anderson, 1991). Such behavior stems from heightened commitment and satisfaction derived from interpersonal interactions fostered by well-meaning bonds within high TMX groups. Individuals in such groups are motivated to behave politely and harmoniously with others, resulting in greater instances of OCBI. Contextual cues indicating high TMX can mitigate the negative effects of age diversity on individual-level OCBs including OCBO and OCBI. Thus, the author proposes the following hypotheses:

*H4a:* TMX will moderate the negative effect of age diversity on OCBO, such that the negative effect of age diversity on OCBO will be mitigated when TMX is high.

*H4b:* TMX will moderate the negative effect of age diversity on OCBI, such that the negative effect of age diversity on OCBI will be mitigated when TMX is high.

**Figure 1:**
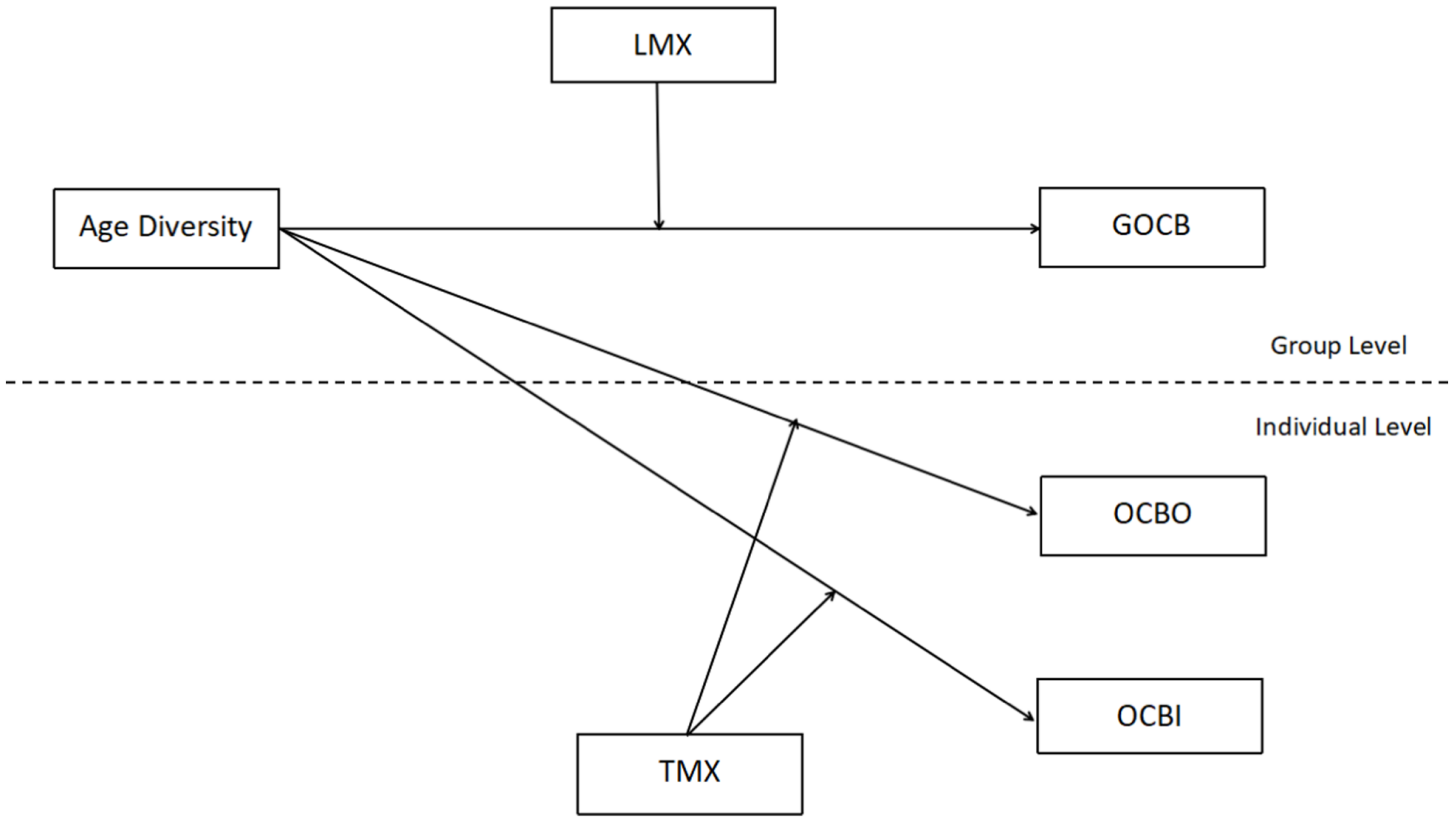
Hypothesized model. *N* = 87 groups; group size, group age and individual age were controlled; GOCB, group organizational citizenship behavior; LMX, leader-member exchange; OCBO, organizational citizenship behavior toward organizations; OCBI, organizational citizenship behavior toward individuals; TMX, team-member exchange.

The comprehensive conceptual model is depicted in Figure 1.

## Method

### Sample and procedures

Multi-wave, multisource data was collected from two state-owned enterprises in China, both of which were heavy industry manufacturing companies. With the assistance of the human resource department (HRD) staff from each firm, the author randomly selected 1,100 individual employees from 102 different groups as initial target respondents. The HRD provided demographic characteristic data. In the first survey (Time 1), each employee participated in an online survey regarding GOCB, TMX and LMX. After a two-week interval, a follow-up survey (Time 2) was conducted where subordinates who had responded to Time 1 were asked to evaluate OCBO and OCBI of each member in their group. Each group member rated their fellow members, and scores for each participant were calculated accordingly. The author excluded cases with fewer than two responding members within groups or missing vital variable data. Out of the total sample of 1,100 employees, 990 (90%) participated in the investigation resulting in a final available sample consisting of 87 work groups with 882 employees (response rate = 89.09%). The method design was multi-source. Because age diversity was calculated based on data provided by HRD rather than through employee questionnaire responses, and OCBO and OCBI were assessed through peer evaluation instead of self-assessment.

Of the 87 groups, 41 belonged to Firm A while the remaining 46 belonged to Firm B. The mean age of all group members was approximately 38.32 (SD = 8.72) years old, while their average tenure was 17.17 (SD = 6.36) years. Among all surveyed group members, the male population accounted for 52.95%. Furthermore, a significant majority of group members (80.95%) held at least a bachelor’s degree.

### Measures

To test the hypotheses, this article used previously validated scales to guarantee reliability and feasibility (Cable and DeRue, 2002; Zhou and George, 2001; Delery, 1998). In terms of questionnaire wording, the author translated the items into Chinese, and then made modifications based on a pretest to improve clarity and facilitate understanding for Chinese respondents. Following established translation procedures (Brislin, 1980), the author back-translated the items into English while striving to maintain fidelity to the original version in order to prevent any distortion. Additionally, a time-lagged multisource design was implemented to reduce potential common source bias.

#### Age diversity

Age diversity was measured in this study using employee data provided by HRD to calculate the age in years. Following the guidelines recommended by Harrison and Klein (2007) for measuring age diversity, within-group standard deviations (SDs) were computed as a metric.

#### GOCB

GOCB was assessed by group individuals using the original four items (*α* = 0.95) developed by Podsakoff et al. (1997). The measurement of GOCB items employed a 7-point Likert scale from 1 (strongly disagree) to 7 (strongly agree). A sample item included “Our team members help each other out if someone falls behind in his/her work.”

#### OCB (OCBO and OCBI)

Using the validated 10-item scale (*α* = 0.95) established by Williams and Anderson (1991). The OCB was assessed by group members using a method in which each member rated their fellow members, and then calculated a score for each participant. This scale encompassed two dimensions of OCB, namely organizational citizenship behavior toward organizations (OCBO) and organizational citizenship behavior toward individuals (OCBI). OCBO consisted of 5 items (*α* = 0.96), with one example being “This employee protects and cherishes the company’s daily supplies and goods.” Additionally, OCBI utilized a 5-item scale (*α* = 0.96), exemplified by “This employee helps others who have heavy workloads”, measured on a 7-point Likert scale (1 = strongly disagree, 7 = strongly agree).

#### LMX

The quality of the relationship between group members and their superiors was assessed using a 7-item scale developed by Scandura and Graen (1984). For instance, one item stated, “My immediate supervisor understands my problems and needs.” Participants rated each item on a seven-point Likert-type scale from 1 (strongly disagree) to 7 (strongly agree). The reliability of the scale was determined through Cronbach’s alpha (*α* = 0.98).

#### TMX

TMX was measured using Seers (1989) 10-item index. The questionnaire included statements such as: “Other team members let me know when I affect their work,” and “Other members recognize my potential.” Employees rated these ten items on a seven-point Likert-type scale from 1 (strongly disagree) to 7 (strongly agree). The Cronbach’s alpha for the ten scales was 0.98.

#### Control variables

This paper controlled for other variables that may exert an influence on this analysis, such as group size (Pearce and Herbik, 2004) and group age (Harrison et al., 1998). Consistent with prior research indicating their potential effects on various processes and outcomes (e.g., Shin and Choi, 2010; Kearney et al., 2009), the author took these factors into consideration. Group size indicated the total number of employees within a specific group. Group age was assessed based on the average years of age among its members in the workgroup. Moreover, previous studies investigating individual OCB highlighted the significance of the individual’s age in shaping OCB (e.g., Organ and Ryan, 2010). Thus, this research also controlled for age at the individual level.

## Results

The means, standard deviations, and correlations of all variables are reported in Table 1. Additionally, for measures collected at the individual level, it is necessary to certify agreement within groups before aggregating to the group level (Harrison et al., 2002). To assess this agreement, this study computed the within-group agreement index (rwg) proposed by James et al. (1993, 1984). Because individual respondents were nested within groups, this research also assessed potential statistical dependence in the data by computing ICC(1) (i.e., an index of within-group variability), and ICC(2) representing between-group variability (Bliese, 2000). For GOCB: 0.99 (rwg), 0.98 (ICC1), 0.99 (ICC2); and for LMX: 0.99 (rwg), 0.72 (ICC1), 0.96 (ICC2). These values provided adequate evidence supporting aggregation of these concepts at the group level.

**Table 1:** Means, standard deviations, and correlations among study variables.

Variables	Mean	SD	Group size	Group age	Age diversity	GOCB	LMX	Individual age	OCBO	OCBI	TMX
Group level											
Group size	11.47	3.41									
Group age	38.32	7.84	−0.06								
Age diversity	3.75	1.43	0.01	0.11**							
GOCB	4.63	1.56	−0.15**	0.04	−0.06	(0.92)					
LMX	4.73	1.68	−0.06	0.04	−0.08*	0.34**	(0.98)				
Individual level											
Individual age	38.32	8.72									
OCBO	4.66	1.70						0.02	(0.96)		
OCBI	4.69	1.62						0.03	0.62**	(0.96)	
TMX	4.64	1.64						0.06	0.42**	0.43**	(0.98)

*N* = 87 groups; group size, group age and individual age were controlled; Cronbach’s alpha internal-consistency reliability coefficients appear in parentheses along the main diagonal; GOCB, group organizational citizenship behavior; LMX, leader-member exchange; OCBO, organizational citizenship behavior toward organizations; OCBI, organizational citizenship behavior toward individuals; TMX, team-member exchange; ^*^p < 0.05; ^**^p < 0.01.

The fit of the scales of GOCB, OCB, LMX, and TMX was evaluated in this article using AMOS 23.0 to determine their distinctiveness. The hypothesized model was compared with alternative models through confirmatory factor analysis (CFA) to scrutinize its distinctiveness. The CFA results demonstrated that the hypothesized five-factor model fits the data well: *χ*^2^ = 934.26, *χ*^2^/*df* (424) = 2.20, CFI = 0.99, TLI = 0.99, RMSEA = 0.04. This study compared this model with plausible alternative models including (1) a four-factor model with OCBO and OCBI combined into a single factor; (2) a three-factor model with OCBO and OCBI combined into a single factor, and GOCB and LMX combined as one construct; (3) a two-factor with OCBO, OCBI and TMX at the individual level combined into a single factor, and group-level factors (i.e., GOCB and LMX) combined as one construct; (4) a one-factor with OCBO, OCBI, TMX, GOCB, and LMX combined as one construct. The expected hypothesized model certified a substantially improved fit compared to relevant alternative models (Table 2).

**Table 2:** Confirmatory factor analysis.

Model	Description	*X* ^2^	*df*	*X*^2^/*df*	CFI	TLI	RMSEA	SRMR
1	Five-factor model^1^	934.26	424	2.20	0.99	0.98	0.04	0.02
2	Four-factor model^2^	4255.44	428	9.94	0.89	0.88	0.10	0.06
3	Three-factor model^3^	7079.37	431	16.43	0.81	0.80	0.13	0.17
4	Two-factor model^4^	12802.14	433	29.80	0.65	0.63	0.18	0.23
5	One-factor model^5^	20723.14	434	47.75	0.43	0.39	0.23	0.23

*N = 87 groups; CFI, Comparative fit index; TLI, Tucker-Lewis index; RMSEA, root mean square error of approximation; SRMR, standardized RMR.*

^1Hypothesized model.^

^2^Organizational citizenship behavior toward organizations (OCBO) and organizational citizenship behavior toward individuals (OCBI) at individual-level combined as one construct.

^3^Organizational citizenship behavior toward organizations (OCBO) and organizational citizenship behavior toward individuals (OCBI) at individual-level combined as one construct, and group organizational citizenship behavior (GOCB) and leader-member exchange (LMX) at group-level combined as one construct.

^4^Organizational citizenship behavior toward organizations (OCBO), organizational citizenship behavior toward individuals (OCBI) and team-member exchange (TMX) at individual-level combined as one construct, and group organizational citizenship behavior (GOCB) and leader-member exchange (LMX) at group-level combined as one construct.

^5^Organizational citizenship behavior toward organizations (OCBO), organizational citizenship behavior toward individuals (OCBI), team-member exchange (TMX), group organizational citizenship behavior (GOCB) and leader-member exchange (LMX) combined as one construct.

The hypothesis 1 regarding the negative effect of age diversity on GOCB was examined through regression analysis in SPSS. The findings proved empirical evidence supporting the hypothesis 1, indicating that age diversity had a detrimental impact on GOCB (*β* = −0.61, *B* = −0.66, *t* = −6.90, *p* < 0.001, *R*^2^ = 0.38, △*F* = 16.96). Moreover, the hypothesis 3 was tested by utilizing Model 1 in PROCESS macro for SPSS (Hayes, 2013), which has gained increasing endorsement for its efficacy in examining moderation effects (e.g., Li et al., 2020). The findings demonstrated a significant interaction between age diversity and LMX on GOCB within a bias-corrected 95% confidence interval (CI): coefficient = 0.44, *t* = 12.37, *p* < 0.001, [0.37, 0.51] (Table 3), supporting Hypothesis 3.

**Figure 2:**
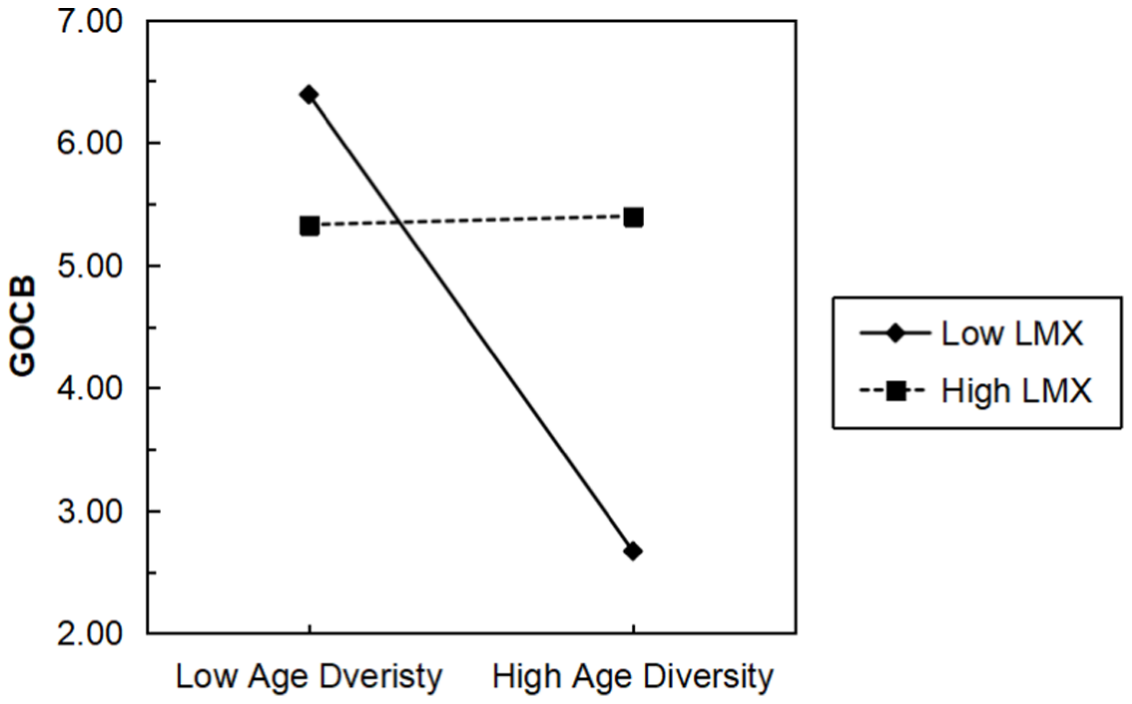
The interactive effect of age diversity and LMX on GOCB. *N* = 87 groups; group size and group age were controlled; GOCB, group organizational citizenship behavior; LMX, leader-member exchange.

The two-way interaction was plotted in this paper based on the model with significant influences, aiming to better interpret the moderating relationships. Following the recommended procedures by Dawson (2014), similar approaches as proposed by Aiken and West (1991) were used to generate a graph of the two-way interaction. Specifically, this study manipulated independent variables one standard deviation above and below their means while holding control variables at their means, and then calculated simple slope tests. The results from these tests revealed a shift in the negative slope of age diversity-GOCB to positive when LMX was high, contrasting with the negative slope observed under low LMX conditions (see Figure 2).

Subsequently, hierarchical linear modeling (HLM; Hox, 2010; Raudenbush et al., 2004) was employed to estimate the hypothesized cross-level effects. To examine research hypotheses 2a, 2b, 4a and 4b, null models were initially run to evaluate the discrepancy of employees’ individual OCBO, OCBI across groups. It is imperative to ascertain whether there is within-group agreement (*σ*^2^) regarding different employees’ perceptions of OCBO and OCBI, and whether these individual perceptions differ between distinct groups (i.e., between-group variation, τ00).

**Table 3:** Results of PROCESS analysis.

	Coefficient	S.E.	*t*	*p*	LLCI	ULCI
Constant	4.91	0.51	9.56	***	3.89	5.93
Group size	−0.02	0.02	−0.94	0.35	−0.06	0.02
Group age	0.01	0.01	0.53	0.60	−0.02	0.03
Age diversity	−0.63	0.06	−11.23	***	0.085	4.541
LMX	0.29	0.06	5.26	***	0.18	0.40
Age diversity*LMX	0.44	0.04	12.37	***	0.37	0.51

*N* = 87 groups; group size and group age were controlled; group organizational citizenship behavior (GOCB) was the outcome variable; LMX, leader-member exchange; Bias-corrected 95% confidence intervals were applied; Mean center was applied for all variables and covariates of this study; ***p < 0.001.

The results presented in Table 4 verified that the value of within-group agreement (*σ*^2^) were: 0.75 (OCBO), 1.08 (OCBI), and the between-group variation (τ00) reached a significant level: OCBO = 2.21 (*χ*^2^ = 2599.39, *df* = 86, *p <* 0.001) and OCBI = 1.60 (*χ*^2^ = 1337.31, *df* = 86, *p* < 0.001). By applying the formula “ICC(1) = τ00/(*σ*^2^ + τ00),” this investigation obtained the values of ICC(1): 0.75 for OCBO and 0.60 for OCBI. Additionally, 0.96 represented the variance of employees’ OCBO, while 0.93 represented the variance of employees’ OCBI from intra-group variance (ICC2). Given that, accurate parameter estimates and significance tests were conducted to account for multilevel structures and non-independent data (Bliese, 2002).

**Table 4:** Hierarchical linear modeling (HLM) analysis of individual outcomes.

	OCBO			OCBI		
	Null model	Model 1	Model 2	Null model	Model 3	Model 4
Level 1						
Individual age		−0.00	−0.00		0.01	0.02
TMX						
Level 2						
Group size		0.01	0.01		0.01	0.02
Group age		0.06**	0.07**		0.07**	0.07**
Age diversity		−0.42**	−0.42**		−0.36**	−0.36**
GOCB						
LMX						
Age Diversity*TMX			0.12*			0.16**
*χ*^2^(df)	2599.39 (86)***			1337.31 (86)***		
*σ* ^2^	0.75			1.08		
τ00	2.21			1.60		
ICC(1)	0.75			0.60		
ICC(2)	0.96			0.93		
Model deviance	2554.08	2541.50	2477.07	2811.91	2795.60	2759.75

*N* = 87 groups; group size, group age and individual age were controlled; in all models, gamma coefficients were presented; OCBO, organizational citizenship behavior toward organizations; OCBI, organizational citizenship behavior toward individuals; TMX, team-member exchange; GOCB, group organizational citizenship behavior; LMX, leader-member exchange; ^**^p < 0.01; ^***^p < 0.001.

The results of hypotheses 2a and 2b indicated that age diversity had negative influences on both OCBO and OCBI. As shown in Table 4, model 1 and model 3 demonstrated significant negative effects of age diversity on OCBO (*γ* = −0.42, *p* < 0.01) and OCBI (*γ* = −0.36, *p* < 0.01). Therefore, these findings provided support for hypotheses 2a and 2b.

**Figure 3:**
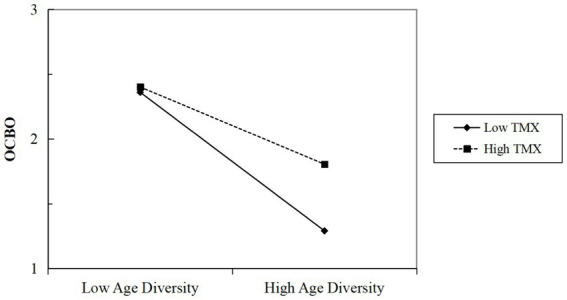
The interactive effect of age diversity and TMX on OCBO. *N* = 87 groups; individual age was controlled; OCBO, individual-level organizational citizenship behavior toward organizations; TMX, team-member exchange.

**Figure 4:**
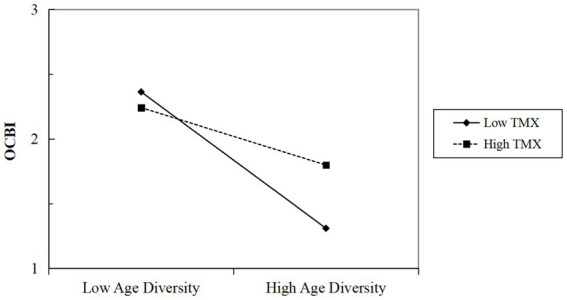
The interactive effect of age diversity and TMX on OCBI. *N* = 87 groups; individual age was controlled; OCBI, individual-level organizational citizenship behavior toward individuals; TMX, team-member exchange.

Additionally, hypotheses 4a and 4b stated that individual-level TMX would attenuate the negative relationships between group-level age diversity and individual-level OCBO and OCBI. This study examined these cross-level moderating effects using slopes-as-outcomes models in HLM (Hofmann et al., 2000). The results from model 2 and model 4 in Table 4 revealed significant interactions between age diversity and TMX on OCBO (*γ* = 0.12, *p* < 0.05) and OCBI (*γ* = 0.16, *p* < 0.01). Figures 3, 4 visually depicted that higher levels of TMX mitigated the negative effects of age diversity on both OCBO and OCBI. Consequently, both hypotheses 4a and 4b were supported.

## Discussion

A growing concern for many organizations is the increasing age diversity. Similar to research on other demographic categories of diversity, the effects of such diversity are often ambiguous, and can be viewed as a “double-edged sword” (Horwitz and Horwitz, 2007, p. 988; for an overview, see Jackson et al., 2003; van Knippenberg and Schippers, 2007). Previous studies have yielded mixed results regarding age diversity (e.g., Ely, 2004; Bunderson and Sutcliffe, 2002; Kilduff et al., 2000), potentially because researchers have overlooked possible mediators or moderators in the relationships between age diversity and its outcomes (Kunze et al., 2011). Therefore, this paper aims to examine contextual factors that influence the relationships between age diversity and its outcomes.

This paper analyzes the relationships between age diversity and its influence on group and individual outcomes, such as GOCB and OCBs. In line with the social categorization (Turner, 1987) and social identity (Tajfel, 1982) theories, age diversity, as a prominent demographic factor (van Prooijen and van Knippenberg, 2000), is more likely to elicit in-group preferences and out-group bias. These detrimentally affect group-level GOCB and individual-level OCBO and OCBI through social categorization processes.

This paper explores the moderating role of LMX in the relationship between age diversity and GOCB. Additionally, this study investigates whether TMX acts as a moderator in the relationships between age diversity and individual-level OCBO and OCBI. The findings support hypotheses of this paper that a high level of LMX serves as a compensatory factor, counterbalancing the negative influence of age diversity on GOCB. When LMX is high, there is a positive relationship between age diversity and GOCB. Since the collective impact of age diversity cannot be simply derived from aggregating individual effects, it provides valuable insights beyond previous research that primarily focuses on understanding how age diversity affects individual outcomes (e.g., Salthouse, 2012; Warr, 1994).

The findings of this study also demonstrate that high TMX serves as a mitigating factor for the adverse effects of age diversity on individual-level OCBO and OCBI. According to the social exchange theory (Blau, 1964), kindness driven by elevated levels of TMX is likely to foster individuals’ sense of identity and compatibility (Chiniara and Bentein, 2017) with their group and fellow members. These positive relationship-oriented interactions, in turn, encourage individuals to reciprocate the favors bestowed upon them by the group and other members through engaging in extra-role actions. This can partially alleviate the negative effects of age diversity on individual behaviors, such as OCBO and OCBI.

Overall, this study adopts a comprehensive and holistic approach to conduct multi-level analyses within the context of an aging labor force, thereby providing valuable insights for research methods in senior labor force studies and promoting scientific inquiry into human resource practices (HRPs) amidst population aging. By examining moderation pathways, this investigation responds to calls for “further develop our understanding of moderating influences that may speak to ways to manage diversity” (van Knippenberg and Mell, 2016, p. 142).

## Theoretical implications

This research contributes to the extant literature in several ways. First, this paper expands upon previous research focused on the effects of age diversity by incorporating the CEM (van Knippenberg et al., 2004). The CEM is a well-developed paradigm that includes boundary conditions and helps to refine age diversity research. Building upon the CEM (van Knippenberg et al., 2004), this study introduces novel boundary factors into age diversity research, namely LMX and TMX. It surveys the interactive influence of age diversity and LMX on GOCB while also exploring the moderating effects of TMX in the relationships between age diversity and OCB. The findings of this investigation claim that within groups characterized by high levels of LMX, age diversity exhibits a positive association with GOCB rather than a negative one. Moreover, high TMX acts as a moderator by mitigating the negative impact of age diversity on OCB. In this sense, this investigation advances understanding of age diversity research and provides a valuable theoretical framework for determining multi-level outcomes.

Second, in contrast to previous research that predominantly focuses on the individual level, this study aims to comprehensively explore OCB at both the group and individual levels, thereby addressing the multi-level nature of this construct. Scholars have identified that GOCB, as a concept at the group level, is distinct from a mere aggregation of individual-level OCB (Shin and Choi, 2010). Empirical findings of this research confirm of the presence of GOCB as a meaningful behavioral domain at the group level. Incorporating GOCB into future research on group behaviors holds significant potential for advancing the understanding in this area. This study contributes to existing literature on OCB by expanding its investigation across both group and individual levels.

Third, this study promotes the advancement of the CEM theory (van Knippenberg et al., 2004) and multi-level OCB by examining their applicability in the context of population aging. Population aging and HRPs have emerged as prominent areas of research within modern organizations. However, there is still a significant gap in integrating these two fields for mutual benefit. To address this gap, this study analyzes HRPs within the macro context of the Age of aging, thereby offering potential insights for further development and enrichment of both HRPs and population aging research.

## Practical implications

Effective management of workplace diversity is crucial for HRPs (Mor Barak, 2022; Triana et al., 2010). Given the global aging context, conducting focused research on age diversity becomes imperative. This paper raises several practical implications for organizations seeking strategies to effectively manage an increasingly diverse workforce in terms of age. Maintaining high levels of TMX is essential in motivating employees to contribute their age-based diverse resources and engage in more extra-role behaviors. Age stereotypes and biases often lead to conflicts and hostility that can divide the group and hinder OCB, thereby impacting overall organizational functioning. A high level of TMX can alleviate competition and animosity arising from age heterogeneity within the group. To this end, it is meaningful to attach importance to high levels of TMX. Establishing an open information-exchange forum could serve as a practical approach by providing an interactive platform where employees from different groups can freely express their opinions and suggest areas for improvement (Wang and Noe, 2010). The implementation of regular group-building activities can serve as an effective strategy to facilitate employee communication and enhance interpersonal relationships among group members.

The findings of this study also suggest that managers should recognize and proactively address the detrimental effects arising from age diversity. It is vital for organizations and groups to establish mechanisms and create environments that foster effective communication and cooperation among group members, particularly when the group consists of distinct age-based social categorization subgroups. Communication and collaboration are advantageous for group members who perceive social support and harmony rather than tension and animosity. Based on the results of this paper, cultivating high LMX is a feasible and valuable approach. The extent to which group members feel disconnected from their leader represents a significant source of frustration and depletion of energy (Clercq, 2021). These negative emotions and alienation from the organization may exacerbate the challenges posed by age diversity, hindering GOCB. As key figures guiding organizational development, managers should make every effort to mitigate such harmful emotions and beliefs within their ranks. Additionally, managers need to cultivate an internal environment where individuals within the group feel comfortable and secure engaging in discussions with others. Groups featured by high levels of LMX exhibit more positive attitudes toward the ingroup compared to those with low LMX, thereby facilitating the establishment of a favorable internal setting while mitigating the adverse influence of age diversity on GOCB.

Notably, it is important to acknowledge and emphasize the significance of leadership behavior, particularly in informal processes. In this regard, managers should act as exemplary role models. Moreover, managers must pay closer attention to the relationships among group employees and between leaders and members. Managers’ endeavors are expected to foster an organizational culture that values and supports harmonious relationships while appreciating age diversity from various perspectives. Implementing diversity training can be a cost-effective strategy for achieving this objective. Additionally, the development of personalized HRPs tailored to age diversity for each employee would be a groundbreaking approach. This strategy ensures a mutually agreeable and harmonious environment, enabling all group members to appreciate the value of age diversity rather than perceiving it as a potential threat.

## Limitations and future research

Despite several significant implications, however, this current study does possess certain limitations that require acknowledgment and interpretation, each of which presents opportunities for future research. First, it is important to consider potential cross-cultural limitations when discussing age composition in group contexts. Generally speaking, “an individualist culture, often found in Western societies, tends to be directed towards the independent role and functions of individual human beings” (Seong and Hong, 2018, p. 15). Conversely, collectivist cultures place a greater emphasis on collective relationships (Triandis, 1995), and are more proactive in venerating senior adults.

Given China’s collectivist nature, it is crucial to carefully assess whether there is an excessively optimistic evaluation for collective behaviors such as GOCB. Furthermore, with the increasing diversification of the workforce, numerous societies, including China, are facing the challenge posed by an expanding generation gap based on age within their workplaces. However, East Asian societies still uphold the enduring tradition of venerating elders which plays a pivotal role in fostering intergenerational cohesion and unity (Sung, 2007; Alston, 1989). The reverence for older individuals as “treasures” prevails in Chinese society due to their extensive life experiences and remarkable professional achievements. This significantly contributes to mitigating age discrimination and exclusion. Therefore, the evaluation of TMX from diverse age groups should consider cultural differences, as they can lead to varying perspectives on the elderly.

Given that, the author advocates for caution in generalizing the results. The findings of this study reveal a positive association between the interaction effect of age diversity and high LMX on GOCB, as well as the mitigating role of high TMX in reducing the negative influence of age diversity on OCB within the relationship-oriented and elder-respected collectivist nature context. However, further investigation is required to ascertain the directionality or strength of these relationships across diverse cultures. Future research should incorporate comparative studies encompassing different cultures to validate the preliminary conclusions drawn from this analysis.

Second, there are several measurement issues. Specifically, this investigation employed a time-lagged design where individual-level OCB ratings were collected from group members two weeks after conducting surveys on GOCB, LMX and TMX survey. This approach helps reduce potential common method bias and strengthens confidence in the validity of the findings. Nevertheless, it is worth noting that this design represents a cross-sectional analysis, which introduces the possibility of reverse causality. Consequently, doubts may emerge concerning the causal relationships predicted by the theoretical model of this research.

Moreover, the self-report measurement possesses adequate rationalization and advantages, as it offers a larger number of instances (Parker and Collins, 2010), and enables better observation of behavioral differences in specific contexts (Lance et al., 1992). Consequently, the data for this analysis was collected through self-reports provided by group employees while excluding demographic statistics obtained from HRD and OCB (the report was provided by colleagues). However, the self-report measure suffers from a significant limitation. It may introduce a risk of common method bias that can affect outcomes (Podsakoff et al., 2003). Hence, the author encourages future research to adopt a longitudinal design and strive to incorporate more objective indicators for exploring and interpreting causal relationships among age diversity, GOCB, OCB, LMX, and TMX.

Third, the focus of this research is on age diversity, an inevitable and essential trend in HRPs’ research on aging. To gain a deeper understanding of the potential implications of age diversity on GOCB and OCB, it is necessary to inspect psychological and social processes that may be at play. In other words, there is a need to clarify the “black box” phenomenon. In addition, since this study solely focuses on the relationships among age diversity, GOCB, OCB, LMX, and TMX, it would be valuable to investigate other intricacies or nuances within HRPs or its architectural framework. Therefore, future research should explore specific mediators, consider similar buffering contexts, and examine additional outcome variables to adequately elucidate the dynamics of HRPs in an aging workforce.

## Conclusion

This paper contributes to existing research by comprehensively analyzing the effects of age diversity on OCB at both the group and individual levels, as well as examining how group age diversity, LMX, and TMX collectively influence multi-level OCB. The findings provide support for the theoretical argument that high LMX can mitigate the detrimental impact of age diversity on GOCB. Similarly, a high level of TMX can alleviate the negative effects caused by age diversity on individual OCBs. In conclusion, these findings emphasize the significance of utilizing both LMX and TMX to counteract any adverse effects of age diversity on OCB at both group and individual levels. This contributes to advancements in HRPs as well as population aging research.

## Data Availability

The raw data supporting the conclusions of this article will be made available by the authors, without undue reservation.

## References

[ref1] Aiken L. S. West S. G. (1991). Multiple regression: Testing and interpreting interactions. Newbury Park, CA: Sage.

[ref2] Alston J. P. (1989). Wa, Guanxi, and Inhwa: managerial principles in Japan, China, and Korea. Bus. Horiz. 32, 26–31. doi: 10.1016/S0007-6813(89)80007-2

[ref3] Bartunek J. M. Krim R. M. Necochea R. Humphries M. (1999). “Sensemaking, sensegiving, and leadership in strategic organizational development” in Advances in qualitative organization research. ed. Wagner J. A. III (Netherlands (NL): Elsevier Science/JAI Pres), 36–71.

[ref4] Blau P. M. (1964). Exchange and power in social life. New York: Wiely.

[ref5] Bliese P. D. (2000). “Within-group agreement, non-independence, and reliability: implications for data aggregation and analysis” in Multilevel theory, research, and methods in organizations: Foundations, extensions, and new directions. eds. Kozlowski S. W. J. Klein K. J. (San Francisco, CA: Jossey-Bass/Pfeiffer), 305–327.

[ref6] Bliese P. D. (2002). “Using multilevel random coefficient modeling in organizational research” in Advances in measurement and data analysis. eds. Drasgow F. Schmitt N. (San Francisco, CA: Jossey-Bass), 401–445.

[ref7] Bommer W. H. Dierdorff E. C. Rubin R. S. (2007). Does prevalence mitigate relevance? The moderating effect of group-level OCB on employee performance. Acad. Manag. J. 50, 1481–1494. doi: 10.5465/amj.2007.28226149

[ref8] Borman W. C. Motowidlo S. J. (1993). “Expanding the criterion domain to include elements of contextual performance” in Personnel selection in organizations. eds. Schmitt N. Borman W. C., and Associates (San Francisco, CA: Jossey-Bass), 71–98.

[ref9] Borman W. Motowidlo S. (1997). A theory of individual differences in task and contextual performance. Hum. Perform. 10, 99–109. doi: 10.1207/s15327043hup1002_3

[ref10] Brislin R. W. (1980). “Translation and content analysis of oral and written material” in Handbook of cross-cultural psychology. eds. Triandis H. C. Berry J. W. (Boston, MA: Allyn & Bacon), 349–444.

[ref11] Brown A. Guttmann R. (2017). Ageing and labour supply in advanced economies. RBA Bullet, 37–46.

[ref12] Bunderson J. S. Sutcliffe K. M. (2002). Comparing alternative conceptualizations of functional diversity in management teams: process and performance effects. Acad. Manag. J. 45, 875–893. doi: 10.2307/3069319

[ref13] Cable D. M. DeRue D. S. (2002). The convergent and discriminant validity of subjective fit perceptions. J. Appl. Psychol. 87, 875–884. doi: 10.1037/0021-9010.87.5.875, PMID: 12395812

[ref14] Chan D. (1998). Functional relations among constructs in the same content domain at different levels of analysis: a typology of composition models. J. Appl. Psychol. 83, 234–246. doi: 10.1037/0021-9010.83.2.234

[ref15] Chattopadhyay P. (1999). Beyond direct and symmetrical effects: the influence of demographic dissimilarity on organizational citizenship behavior. Acad. Manag. J. 42, 273–287. doi: 10.2307/256919

[ref16] Chen X.-P. Lam S. S. K. Naumann S. E. Schaubroeck J. (2005). Group citizenship behaviour: conceptualization and preliminary tests of its antecedents and consequences. Manag. Organ. Rev. 1, 273–300. doi: 10.1111/j.1740-8784.2005.00012.x

[ref17] Chen X. P. Lam S. S. K. Schaubroeck J. Naumann S. (2002). Group organizational citizenship behavior: a conceptualization and preliminary test of its antecedents and consequences. Acad. Manag. Proc. 1, 273–300. doi: 10.1111/j.1740-8784.2005.00012.x

[ref18] Chiniara M. Bentein K. (2017). The servant leadership advantage: when perceiving low differentiation in leader-member relationship quality influences team cohesion, team task performance and service OCB. Leadersh. Q. 29, 333–345. doi: 10.1016/j.leaqua.2017.05.002

[ref19] Choi J. N. (2009). Collective dynamics of citizenship behaviour: what group characteristics promote group-level helping? J. Manag. Stud. 46, 1396–1420. doi: 10.1111/j.1467-6486.2009.00851.x

[ref20] Choi J. N. Sy T. (2010). Group-level organizational citizenship behavior: effects of demographic faultlines and conflict in small work groups. J. Organ. Behav. 31, 1032–1054. doi: 10.1002/job.661

[ref21] Clercq D. D. (2021). Organizational disidentification and change-oriented citizenship behavior. Eur. Manag. J. 40, 90–102. doi: 10.1016/j.emj.2021.02.002, PMID: 39947943

[ref22] Dawson J. F. (2014). Moderation in management research: what, why, when and how. J. Bus. Psychol. 29, 1–19. doi: 10.1007/s10869-013-9308-7

[ref23] De Janasz D. Dowd K. O. Schneider B. Z. (2015). Interpersonal skills in organisation. New York, NY: McGraw-Hill Education.

[ref24] Delery J. E. (1998). Issues of fit in strategic human resource management: implications for research. Hum. Resour. Manag. Rev. 8, 289–309. doi: 10.1016/S1053-4822(98)90006-7

[ref25] Dineen B. R. Lewicki R. J. Tomlinson E. (2006). Supervisory guidance and behavioral integrity: relationships with employee citizenship and deviant behavior. J. Appl. Psychol. 91, 622–635. doi: 10.1037/0021-9010.91.3.622, PMID: 16737359

[ref26] Ehrhart M. G. (2004). Leadership and procedural justice climate as antecedents of unit-level organizational citizenship behavior. Pers. Psychol. 57, 61–94. doi: 10.1111/j.1744-6570.2004.tb02484.x

[ref27] Eisenberger R. Armeli S. Rexwinkel B. Lynch P. Rhoades L. (2001). Reciprocation of perceived organizational support. J. Appl. Psychol. 86, 42–51. doi: 10.1037/0021-9010.86.1.42, PMID: 11302232

[ref28] Eisenberger R. Stinglhamber F. Vandenberghe C. Sucharski I. L. Rhoades L. (2002). Perceived supervisor support: contributions to perceived organizational support and employee retention. J. Appl. Psychol. 87, 565–573. doi: 10.1037/0021-9010.87.3.565, PMID: 12090614

[ref29] Ely R. J. (2004). A field study of group diversity, participation in diversity education programs, and performance. J. Organ. Behav. 25, 755–780. doi: 10.1002/job.268

[ref30] Farouk A. M. (2002). Elements of justice and organizational commitment: The impact of leader-member exchange. Unpublished MBA thesis, Penang, Malaysia: University Science Malaysia.

[ref31] Fiske S. T. (2017). Prejudices in cultural contexts: shared stereotypes (gender, age) versus variable stereotypes (race, ethnicity, religion). Perspect. Psychol. Sci. 12, 791–799. doi: 10.1177/1745691617708204, PMID: 28972839 PMC5657003

[ref32] Gerstner C. R. Day D. V. (1997). Meta-analytic review of leader member exchange theory: correlates and construct issues. J. Appl. Psychol. 82, 827–844. doi: 10.1037/0021-9010.82.6.827

[ref33] Gomez C. Rosen B. (2001). The leader-member exchange as a link between managerial trust and employee empowerment. Group Org. Manag. 26, 53–69. doi: 10.1177/1059601101261004

[ref34] Gouldner A. W. (1960). The norm of reciprocity: a preliminary statement. Am. Sociol. Rev. 25, 161–178. doi: 10.2307/2092623

[ref35] Graen G. B. Uhl-Bien M. (1995). Relationship-based approach to leadership: development of leader- member exchange (LMX) theory of leadership over 25 years: applying a multi-level multi-domain perspective. Leader. Quart. 6, 219–247. doi: 10.1016/1048-9843(95)90036-5

[ref36] Graham J. W. (1991). An essay on organizational citizenship behavior. Empl. Responsib. Rights J. 4, 249–270. doi: 10.1007/BF01385031

[ref37] Hackman J. R. (1992). “Team influences on individuals in organizations” in Handbook of industrial and organizational psychology. eds. Dunnette M. D. Hough L. M. (Palo Alto, CA: Consulting Psychologists Press), 199–267.

[ref38] Halbesleben J. R. (2010). “A meta-analysis of work engagement: relationships with burnout, demands, resources, and consequences” in Work engagement: A handbook of essential theory and research. eds. Bakker A. B. Leiter M. P. (Psychology Press), 102–117.

[ref39] Harrison D. A. Klein K. J. (2007). What’s the difference? Diversity constructs as separation, variety, or disparity in organizations. Acad. Manag. Rev. 32, 1199–1228. doi: 10.5465/amr.2007.26586096

[ref40] Harrison D. A. Price K. H. Bell M. P. (1998). Beyond relational demography: time and the effects of surface-and deep-level diversity on workgroup cohesion. Acad. Manag. J. 41, 96–107. doi: 10.2307/256901

[ref41] Harrison D. A. Price K. H. Gavin J. A. Florey A. T. (2002). Time, teams, and task performance: changing effects of surface-and deep-level diversity on group functioning. Acad. Manag. J. 45, 1029–1045. doi: 10.2307/3069328

[ref42] Hayes A. F. (2013). Introduction to mediation, moderation, and conditional process analysis: A regression-based approach. New York, NY: The Guilford Press.

[ref43] Hofmann D. A. Griffin M. A. Gavin M. B. (2000). “The application of hierarchical linear modeling to organizational research” in Multilevel theory, research and methods in organizations: Foundations, extensions, and new directions. eds. Klein K. J. Kozlowski S. W. J. (San Francisco: Jossey-Bass), 467–511.

[ref44] Hogg M. A. Terry D. J. (2000). Social identity and self-categorization processes in organizational contexts. Acad. Manag. Rev. 25, 121–140. doi: 10.2307/259266

[ref45] Holmes J. G. (1981). “The exchange process in close relationships: Microbahviour and macromotives” in The justice motive in social behaviour. eds. Lerner M. J. Lerner S. C. (New York: Plenum), 261–284. doi: 10.1007/978-1-4899-0429-4_12

[ref46] Horwitz S. K. Horwitz I. B. (2007). The effects of team diversity on team outcomes: a meta-analytic review of team demography. J. Manag. 33, 987–1015. doi: 10.1177/0149206307308587

[ref47] Hox J. J. (2010). Multilevel analysis: Techniques and applications. New York, NY: The Routledge Press.

[ref48] Ilies R. Nahrgang J. D. Morgeson F. P. (2007). Leader-member exchange and citizenship behaviors: a meta-analysis. J. Appl. Psychol. 92, 269–277. doi: 10.1037/0021-9010.92.1.269, PMID: 17227168

[ref49] Jackson S. E. Joshi A. Erhardt N. L. (2003). Recent research on team and organizational diversity: SWOT analysis and implications. J. Manag. 29, 801–830. doi: 10.1016/S0149-2063(03)00080-1

[ref50] James L. R. Demaree R. G. Wolf G. (1984). Estimating within-group interrater reliability with and without response bias. J. Appl. Psychol. 69, 85–98. doi: 10.1037/0021-9010.69.1.85

[ref51] James L. R. Demaree R. G. Wolf G. (1993). Rwg: an assessment of within-group interrater agreement. J. Appl. Psychol. 78, 306–309. doi: 10.1037/0021-9010.78.2.306

[ref52] Johns G. (2006). The essential impact of context on organizational behavior. Acad. Manag. Rev. 31, 386–408. doi: 10.5465/amr.2006.20208687, PMID: 39912352

[ref53] Jukka T. (2021). Top management team demography and firm operating performance: a path analysis. J. Strateg. Manag. 14, 19–34. doi: 10.1108/JSMA-12-2019-0224, PMID: 35579975

[ref54] Kearney E. Gebert D. Voelpel S. C. (2009). When and how diversity benefit teams: the importance of team member’s need for cognition. Acad. Manag. J. 52, 581–598. doi: 10.5465/amj.2009.41331431

[ref55] Kidwell R. E. Jr. Mossholder K. W. Bennett N. (1997). Cohesiveness and organizational citizenship behavior: a multilevel analysis using work groups and individuals. J. Manag. 23, 775–793. doi: 10.1177/014920639702300605

[ref56] Kilduff M. Angelmar R. Mehra A. (2000). Top management-team diversity and firm performance: examining the role of cognitions. Organ. Sci. 11, 21–34. doi: 10.1287/orsc.11.1.21.12569

[ref57] Kunze F. Boehm S. A. Bruch H. (2011). Age diversity, age discrimination climate and performance consequences-a cross organizational study. J. Organ. Behav. 32, 264–290. doi: 10.1002/job.698

[ref58] Kunze F. Bruch H. (2010). Age-based faultlines and perceived productive energy: the moderation of transformational leadership. Small Group Res. 41, 593–620. doi: 10.1177/1046496410366307

[ref59] Lance C. E. Teachout M. S. Donnelly T. M. (1992). Specification of the criterion construct space: an application of hierarchical confirmatory factor analysis. J. Appl. Psychol. 77, 437–452. doi: 10.1037/0021-9010.77.4.437

[ref60] Leonard D. Straus S. (1996). Putting your company’s whole brain to work. Harv. Bus. Rev. 75, 110–121. 10168332

[ref61] LePine J. A. van Dyne L. (2001). Peer responses to low performers: an attributional model of helping in the context of groups. Acad. Manag. Rev. 26, 67–84. doi: 10.2307/259395

[ref62] Levi D. (2017). Group dynamics for teams. Los Angeles, CA: Sage.

[ref078] Li Y. Burmeister A. Wang M. Alterman V. Robinson S. (2020). Leveraging age diversity for organizational performance: an intellectual capital perspective. J. Appl. Psychol. 106, 71–91. doi: 10.1037/apl0000497 32202816

[ref64] Li J. Chu C. W. L. Lam K. C. K. Liao S. (2011). Age diversity and firm performance in an emerging economy: implications for cross cultural human resource management. Hum. Resour. Manag. 50, 247–270. doi: 10.1002/hrm.20416

[ref65] Liden R. C. Sparrowe R. T. Wayne S. J. (1997). “Leader-member exchange theory: the past and potential for the future” in Research in personnel and human resources management. ed. Ferris G. R. (Elsevier Science/JAI Press), 47–119.

[ref66] Loretto W. Lain D. Vickerstaff S. Fuertes V. (2013). Extending working lives: Age management in SMEs. Empl. Relat. 35, 272–293. doi: 10.1108/01425451311320477, PMID: 35579975

[ref67] Loretto W. Vickerstaff S. (2013). The domestic and gendered context for retirement. Hum. Relat. 66, 65–86. doi: 10.1177/0018726712455832

[ref68] Luksyte A. Avery D. R. Johnson L. U. Crepeau L. Parker S. K. Wang Y. L. (2022). Age diversity in teams: examining the impact of the least agreeable member. J. Organ. Behav. 43, 546–565. doi: 10.1002/job.2570

[ref69] Maitlis S. Lawrence T. B. (2007). Triggers and enablers of sensegiving in organizations. Acad. Manag. J. 50, 57–84. doi: 10.5465/amj.2007.24160971

[ref70] Mamman A. Kamoche K. Bakuwa R. (2012). Diversity, organizational commitment and organizational citizenship behavior: an organizing framework. Hum. Resour. Manag. Rev. 22, 285–302. doi: 10.1016/j.hrmr.2011.12.003

[ref71] Marotto M. Roos J. Victor B. (2010). Collective virtuosity in organizations: a study of peak performance in an orchestra. J. Manag. Stud. 44, 388–413. doi: 10.1111/j.1467-6486.2007.00682.x, PMID: 39948053

[ref72] Meister J. C. Willyerd K. (2010). Mentoring Millennials. Harv. Bus. Rev. 88, 68–72. doi: 10.1007/s10726-010-9192-8, PMID: 20429252

[ref73] Messick D. Brewer M. (1983). “Solving social dilemmas: a review” in Review of personality and social psychology. eds. Wheeler L. Shaver P. (Beverly Hills, CA: Sage), 11–44.

[ref74] Mitchell R. Gu J. Boyle B. (2024). The impact of leader member exchange quality and differentiation on counterproductive and citizenship behavior in health care teams. Health Care Manag. Rev. 49, 86–93. doi: 10.1097/HMR.0000000000000394, PMID: 38393981

[ref75] Mor Barak M. E. (2022). Managing diversity: Toward a globally inclusive workplace. 5th Edn: Sage Publications.

[ref76] Morgeson F. P. Hofmann D. A. (1999). The structure and function of collective constructs: implications for multilevel research and theory development. Acad. Manag. Rev. 24, 249–265. doi: 10.2307/259081

[ref77] Morrison E. W. Phelps C. C. (1999). Taking charge at work: extra-role efforts to initiate workplace change. Acad. Manag. J. 42, 403–419. doi: 10.2307/257011

[ref78] Nagarajan N. R. Wada M. Fang M. L. Sixsmith A. (2019). Defining organizational contributions to sustaining an ageing workforce: a bibliometric review. Eur. J. Ageing 16, 337–361. doi: 10.1007/s10433-019-00499-w, PMID: 31543728 PMC6728406

[ref79] Ng E. S. Parry E. (2016). “Multigenerational research in human resource management” in Research in personnel and human resources management. eds. Buckley M. R. Halbesleben J. R. B. Wheeler A. R. (Bingley: Emerald Group Publishing), 1–41. doi: 10.1108/S0742-730120160000034008

[ref80] North M. S. Fiske S. T. (2015). Modern attitudes toward older adults in the aging world: a cross-cultural meta-analysis. Psychol. Bull. 141, 993–1021. doi: 10.1037/a0039469, PMID: 26191955

[ref81] Northcraft G. B. Polzer J. T. Neale M. A. Kramer R. M. (1995). “Diversity in work teams: research paradigms for a changing workplace” in Diversity in work teams: Research paradigms for a changing workplace. eds. Jackson S. E. Ruderman M. N. (Washington, DC, US: American Psychological Association), 69–96. doi: 10.1037/10189-003

[ref82] Organ D. W. (1988). Organizational citizenship behavior: The good soldier syndrome. Lexington, MA: Lexington Books.

[ref83] Organ D. W. Paine J. B. (1999). “A new kind of performance for industrial and organizational psychology: recent contributions to the study of organizational citizenship behavior” in International review of industrial and organizational psychology. eds. Cooper C. L. Robertson I. T. (Chichester, UK: American Ethnological Press), 337–368.

[ref84] Organ D. W. Podsakoff P. M. Mac Kenzie S. B. (2006). Organizational citizenship behavior: Its nature, antecedents, and consequences. Thousand Oaks, CA: Sage.

[ref85] Organ D. W. Ryan K. (2010). A meta-analytic review of attitudinal and dispositional predictors of organizational citizenship behavior. Pers. Psychol. 48, 775–802. doi: 10.1111/j.1744-6570.1995.tb01781.x, PMID: 39948053

[ref86] Orpen C. (1994). The effects of organizational and individual career management on career success. Int. J. Manpow. 15, 27–37. doi: 10.1108/01437729410053617

[ref87] Parker S. K. Collins C. G. (2010). Taking stock: integrating and differentiating multiple proactive behaviors. J. Manag. 36, 633–662. doi: 10.1177/0149206308321554

[ref88] Pearce C. L. Herbik P. A. (2004). Citizenship behavior at the team level of analysis: the effects of team leadership, team commitment, perceived team support, and team size. J. Soc. Psychol. 144, 293–310. doi: 10.3200/SOCP.144.3.293-310, PMID: 15168430

[ref89] Pelled L. H. Eisenhardt K. M. Xin K. R. (1999). Exploring the black box: an analysis of work group diversity, conflict, and performance. Adm. Sci. Q. 44, 1–28. doi: 10.2307/2667029

[ref90] Podsakoff P. M. Ahearne M. Mac Kenzie S. B. (1997). Organizational citizenship behavior and the quantity and quality of work group performance. J. Appl. Psychol. 82, 262–270. doi: 10.1037/0021-9010.82.2.262, PMID: 9109284

[ref91] Podsakoff P. M. Mac Kenzie S. B. Lee J. Y. Podsakoff N. P. (2003). Common method biases in behavioral research: a critical review of the literature and recommended remedies. J. Appl. Psychol. 88, 879–903. doi: 10.1037/0021-9010.88.5.879, PMID: 14516251

[ref92] Podsakoff P. M. Mac Kenzie S. B. Moorman R. H. Fetter R. (1990). Transformational leader behaviors and their effects on followers’ trust in leader, satisfaction, and organizational citizenship behaviors. Leader. Quart. 1, 107–142. doi: 10.1016/1048-9843(90)90009-7

[ref93] Podsakoff N. P. Whiting S. W. Podsakoff P. M. Blume B. D. (2009). Individual and organizational level consequences of organizational citizenship behaviors: a meta-analysis. J. Appl. Psychol. 94, 122–141. doi: 10.1037/a0013079, PMID: 19186900

[ref94] Profili S. Sammarra A. Innocenti L. (2017). Age diversity in the workplace: An organizational perspective. Bingley, UK: Emerald Publishing Limited.

[ref95] Raudenbush S. W. Bryk A. S. Congdon R. (2004). HLM 6 for windows [computer software]. Lincolnwood, IL: Scientific Software International.

[ref96] Salthouse T. (2012). Consequences of age-related cognitive declines. Annu. Rev. Psychol. 63, 201–226. doi: 10.1146/annurev-psych-120710-100328, PMID: 21740223 PMC3632788

[ref97] Scandura T. Graen G. B. (1984). Moderating effects of initial leader-member exchange status on the effects of a leadership intervention. J. Appl. Psychol. 69, 428–436. doi: 10.1037/0021-9010.69.3.428

[ref98] Scheuer C. L. Loughlin C. (2019). The moderating effects of status and trust on the performance of age-diverse work groups. Evid. Based HRM 7, 56–74. doi: 10.1108/EBHRM-01-2018-0008

[ref99] Scheuer C. L. Loughlin C. (2021). Seizing the benefits of age diversity: could empowering leadership be the answer? Leader. Organiz. Dev. J. 42, 495–515. doi: 10.1108/LODJ-12-2019-0516

[ref100] Schneid M. Isidor R. Steinmetz H. Kabst R. (2016). Age diversity and team outcomes: a quantitative review. J. Manag. Psychol. 31, 2–17. doi: 10.1108/JMP-07-2012-0228

[ref101] Seers A. (1989). Team-member exchange quality: a new construct for role-making research. Organ. Behav. Hum. Decis. Process. 43, 118–135. doi: 10.1016/0749-5978(89)90060-5

[ref102] Seong J. Y. Choi J. N. (2019). Is person–organization fit beneficial for employee creativity? Moderating roles of leader–member and team–member exchange quality. Hum. Perform. 32, 129–144. doi: 10.1080/08959285.2019.1639711

[ref103] Seong J. Y. Hong D. S. (2018). Age diversity, group organisational citizenship behaviour, and group performance: exploring the moderating role of charismatic leadership and participation in decision-making. Hum. Resour. Manag. J. 28, 621–640. doi: 10.1111/1748-8583.12197

[ref104] Shin Y. H. Choi J. N. (2010). What makes a group of good citizens? The role of perceived group-level fit and critical psychological states in organizational teams. J. Occup. Organ. Psychol. 83, 531–552. doi: 10.1348/096317909X440233

[ref105] Shin S. J. Zhou J. (2007). When is educational specialization heterogeneity related to creativity in research and development teams? Transformational leadership as a moderator. J. Appl. Psychol. 92, 1709–1721. doi: 10.1037/0021-9010.92.6.1709, PMID: 18020807

[ref106] Somech A. Drach-Zahavy A. (2004). Exploring organizational citizenship behavior from an organizational perspective: the relationship between organizational learning and organizational citizenship behavior. J. Occup. Organ. Psychol. 77, 281–298. doi: 10.1348/0963179041752709

[ref107] Staw B. M. Sutton R. I. (1993). “Macro organizational psychology” in Social psychology in organizations: Advances in theory and research. ed. Murnighan J. K. (Englewood Cliffs, NJ: Prentice-Hal), 388–400.

[ref108] Stone D. L. Tetrick L. E. (2013). Understanding and facilitating age diversity in organizations. J. Manag. Psychol. 28, 725–728. doi: 10.1108/JMP-07-2013-0226

[ref109] Sung K. (2007). Respect and care for the elderly: The east Asian way. Lanham, MD: University Press of America.

[ref110] Tajfel H. (1982). Social psychology of intergroup relations. Annu. Rev. Psychol. 33, 1–39. doi: 10.1146/annurev.ps.33.020182.000245

[ref111] Takeuchi R. Bolino M. Lin C. C. (2015). Too many motives? The interactive effects of multiple motives on organizational citizenship behavior. J. Appl. Psychol. 100, 1239–1248. doi: 10.1037/apl0000001, PMID: 25198096

[ref112] Taylor S. E. Sherman D. K. Kim H. S. Jarcho J. Takagi K. Dunagan M. S. (2004). Culture and social support: who seeks it and why? J. Pers. Soc. Psychol. 87, 354–362. doi: 10.1037/0022-3514.87.3.354 15382985

[ref113] Tempest S. Barnatt C. Coupland C. (2002). Grey advantage: new strategies for the old. Long Range Plan. 35, 475–492. doi: 10.1016/S0024-6301(02)00102-4

[ref114] Triana M. C. García M. F. Colella A. (2010). Managing diversity: how organizational efforts to support diversity moderate the effects of perceived racial discrimination on affective commitment. Pers. Psychol. 63, 817–843. doi: 10.1111/j.1744-6570.2010.01189.x

[ref115] Triandis H. C. (1995). Individualism and collectivism. Boulder, CO: Westview Press.

[ref116] Turner J. C. (1987). Discovering the social group: A self-categorization theory. Oxford, England: Basil Blackwell.

[ref117] United Nations, Department of Economic and Social Affairs, Population Division. (2015). World Population Ageing 2015 (ST/ESA/SER.A/390). United Nations.

[ref118] van Dijk H. van Engen M. L. van Knippenberg D. (2012). Defying conventional wisdom: a meta-analytical examination of the differences between demographic and job-related diversity relationships with performance. Organ. Behav. Hum. Decis. Process. 119, 38–53. doi: 10.1016/j.obhdp.2012.06.003

[ref119] van Knippenberg D. De Dreu C. K. W. Homan A. C. (2004). Work group diversity and group performance: an integrative model and research agenda. J. Appl. Psychol. 89, 1008–1022. doi: 10.1037/0021-9010.89.6.1008, PMID: 15584838

[ref120] van Knippenberg D. Mell J. N. (2016). Past, present, and potential future of team diversity research: from compositional diversity to emergent diversity. Organ. Behav. Hum. Decis. Process. 136, 135–145. doi: 10.1016/j.obhdp.2016.05.007

[ref121] van Knippenberg D. Schippers M. C. (2007). Work group diversity. Annu. Rev. Psychol. 58, 515–541. doi: 10.1146/annurev.psych.58.110405.085546, PMID: 16903805

[ref122] van Prooijen J. W. van Knippenberg D. (2000). Individuation or depersonalization: the influence of personal status position. Group Process. Intergroup Relat. 3, 63–77. doi: 10.1177/1368430200031004

[ref123] Vickerstaff S. (2010). Older workers: the “unavoidable obligation” of extending our working lives? Sociol. Compass 4, 869–879. doi: 10.1111/j.1751-9020.2010.00322.x

[ref124] Wang S. Noe R. A. (2010). Knowledge sharing: a review and directions for future research. Hum. Resour. Manag. Rev. 20, 115–131. doi: 10.1016/j.hrmr.2009.10.001

[ref125] Warr P. (1994). “Age and job performance” in Work and ageing: A European perspective. eds. Snel J. Cremer R. (London, UK: Taylor and Francis), 309–325.

[ref126] Wegge J. Meyer B. (2020). Age diversity and age-based faultlines in teams: understanding a Brezel phenomenon requires a Brezel theory. Work Aging Retire. 6, 8–14. doi: 10.1093/workar/waz017

[ref127] Wheaton F. Crimmins E. M. (2013). “The demography of aging and retirement” in The Oxford handbook of retirement. ed. Wang M. (New York, NY: Oxford University Press), 22–41.

[ref128] Williams L. J. Anderson S. E. (1991). Job satisfaction and organizational commitment as predictors of organizational citizenship and in-role behaviors. J. Manag. 17, 601–617. doi: 10.1177/014920639101700305

[ref129] Zhou J. George J. M. (2001). When job dissatisfaction leads to creativity: encouraging the expression of voice. Acad. Manag. J. 44, 682–696. doi: 10.2307/3069410

